# ID 263 - Boletins Informativos do Nats-HUJM

**DOI:** 10.5327/2237-9622.2025.v34s1.263

**Published:** 2025-11-25

**Authors:** Jéssica Weis Bonfanti, Helder Cassio de Oliveira, Letícia Rossetto da Silva Cavalcante

## Abstract

**Introdução:**

Os Núcleos de Avaliação de Tecnologias em Saúde (Nats) foram instituídos nos hospitais de ensino de todo o País com o intuito de introduzir a cultura de Avaliação de Tecnologias em Saúde (ATS) nos hospitais. O Nats é o responsável por realizar ATS por meio da utilização de evidências científicas capazes de auxiliar o gestor hospitalar na tomada de decisão quanto à incorporação, à alteração ou à exclusão de tecnologias, bem como promover o uso apropriado e a racionalidade técnica na alocação de recursos. Além disso, possui o papel fundamental de introduzir e promover a cultura da Prática em Saúde Baseada em Evidências (PSBE) na rotina dos profissionais de saúde. No entanto, a ATS ainda não se apresenta como uma atividade que faça parte da gestão hospitalar atualmente. A falta de cultura de ATS nas instituições gera um grande obstáculo, pois os profissionais da saúde, de maneira geral, desconhecem o tema, mesmo após todos os anos de esforços empreendidos para sua disseminação. Sendo assim, a criação de um Nats não constitui ação suficiente para a difusão da cultura de ATS, havendo fragilidade na influência do núcleo no processo de tomada de decisão clínico e gerencial em hospitais. Dessa forma, a criação dos boletins informativos do Núcleo de Avaliação de Tecnologias em Saúde do Hospital Universitário Júlio Müller (Nats-HUJM) faz parte de um projeto chamado "Desenvolvimento da cultura de avaliação de tecnologias em saúde entre profissionais e gestores de um hospital universitário", que visa ao desenvolvimento de ações que estimulem a cultura do uso da ATS pelos profissionais do HUJM na tomada de decisão. Os objetivos dos boletins informativos são divulgar informações básicas sobre ATS, comunicar as novidades da Comissão Nacional de Incorporação de Tecnologias no SUS (Conitec), incentivar a participação dos profissionais nas decisões do SUS e fortalecer a cultura de ATS no hospital. Para que essa ação não seja negligenciada com o passar do tempo, foi estabelecida uma meta institucional da Unidade de Gestão da Inovação Tecnológica em Saúde (UGITS) para a publicação de, no mínimo, quatro boletins informativos anuais à comunidade hospitalar. Os boletins são divulgados na intranet institucional e disponibilizados tanto no site da unidade quanto nos grupos de mensagens instantâneas institucionais. Cada boletim possui um tema geral que introduz conceitos importantes para a ATS. No ano de 2024, três boletins já foram publicados, os quais abordaram temas como conceitos de tecnologias em saúde e ATS, o Nats e suas funções, e a Conitec e as consultas públicas. Para o presente congresso, a apresentação desta proposta será por meio de comunicação oral expositiva com projeção de multimídia.

**Método:**

A apresentação acontecerá em três etapas. No primeiro momento, será explicada brevemente a justificativa sobre a criação dos boletins. Depois, serão exibidos os três boletins que já foram divulgados na instituição, que abordam os seguintes temas: tecnologias em saúde e ATS, Nats e suas funções, e Conitec e consultas públicas. Ao final, serão discutidas as perspectivas futuras para continuidade das divulgações dos boletins. Será necessário monitor e passador de eslaides com .

